# L-carnitine prevents lenvatinib-induced muscle toxicity without impairment of the anti-angiogenic efficacy

**DOI:** 10.3389/fphar.2023.1182788

**Published:** 2023-04-06

**Authors:** Zheng Jing, Tomohiro Iba, Hisamichi Naito, Pingping Xu, Jun-ichi Morishige, Naoto Nagata, Hironao Okubo, Hitoshi Ando

**Affiliations:** ^1^ Department of Cellular and Molecular Function Analysis, Graduate School of Medical Sciences, Kanazawa University, Kanazawa, Japan; ^2^ Department of Vascular Physiology, Graduate School of Medical Sciences, Kanazawa University, Kanazawa, Japan; ^3^ Department of Gastroenterology, Juntendo University Graduate School of Medicine, Bunkyō, Tokyo, Japan

**Keywords:** angiogenesis, carnitine, lenvatinib, protein synthesis, skeletal muscle, tyrosine kinase inhibitors

## Abstract

Lenvatinib is an oral tyrosine kinase inhibitor that acts on multiple receptors involved in angiogenesis. Lenvatinib is a standard agent for the treatment of several types of advanced cancers; however, it frequently causes muscle-related adverse reactions. Our previous study revealed that lenvatinib treatment reduced carnitine content and the expression of carnitine-related and oxidative phosphorylation (OXPHOS) proteins in the skeletal muscle of rats. Therefore, this study aimed to evaluate the effects of L-carnitine on myotoxic and anti-angiogenic actions of lenvatinib. Co-administration of L-carnitine in rats treated with lenvatinib for 2 weeks completely prevented the decrease in carnitine content and expression levels of carnitine-related and OXPHOS proteins, including carnitine/organic cation transporter 2, in the skeletal muscle. Moreover, L-carnitine counteracted lenvatinib-induced protein synthesis inhibition, mitochondrial dysfunction, and cell toxicity in C2C12 myocytes. In contrast, L-carnitine had no influence on either lenvatinib-induced inhibition of vascular endothelial growth factor receptor 2 phosphorylation in human umbilical vein endothelial cells or angiogenesis in endothelial tube formation and mouse aortic ring assays. These results suggest that L-carnitine supplementation could prevent lenvatinib-induced muscle toxicity without diminishing its antineoplastic activity, although further clinical studies are needed to validate these findings.

## 1 Introduction

Lenvatinib is a small-molecule tyrosine kinase inhibitor (TKI) that targets multiple receptors, including vascular endothelial growth factor receptors (VEGFRs) 1–3, fibroblast growth factor receptors (FGFRs) 1–4, and platelet-derived growth factor receptor (PDGFR) α and β (Matsui et al., 2008). Owing to its potent antineoplastic activity, lenvatinib has become a standard agent for the treatment of several types of advanced cancers such as hepatocellular carcinoma (HCC), differentiated thyroid carcinoma, and renal cell carcinoma (Motzer et al., 2022). However, like other TKIs, lenvatinib often causes adverse drug reactions such as hypertension, diarrhea, proteinuria, and fatigue, which may lead to dose interruption or discontinuation (Kudo et al., 2018), (Kim et al., 2021). In particular, skeletal muscle loss has gained attention as a side effect associated with prognosis (Rinninella et al., 2020).

L-Carnitine is a quaternary ammonium compound derived from dietary sources and endogenous biosynthesis. L-Carnitine homeostasis reflects the balance among intestinal absorption, biosynthesis in several tissues, including the liver, kidney, and brain, renal reabsorption, and muscular storage (Evans and Fornasini, 2003), (Longo et al., 2016), and is maintained by the carnitine/organic cation transporter (OCTN) 2 expressed in these tissues (Tamai, 2013). L-Carnitine facilitates the transport of long-chain fatty acids into the mitochondria for subsequent β-oxidation through the carnitine palmitoyltransferase (CPT) system; acyl-CoAs are converted to acylcarnitines by CPT1 located on the mitochondrial outer membrane, which are translocated across the inner membrane by carnitine/acylcarnitine translocase (CACT) and reconverted to acyl-CoAs by CPT2 located on the inner membrane (Evans and Fornasini, 2003), (Longo et al., 2016). Mitochondrial fatty acid β-oxidation is a key metabolic pathway for energy production, particularly in the heart and skeletal muscle (Bartlett and Eaton, 2004). However, cardiac and skeletal muscles are not capable of synthesizing L-carnitine (Evans and Fornasini, 2003). Therefore, the supply of an adequate amount of L-carnitine is essential for functioning of these tissues.

Our previous study demonstrated that a 14-day daily administration of lenvatinib to rats reduced OCTN2 protein levels in the skeletal muscle and intestine, but not in the kidney, and consequently decreased L-carnitine content in the skeletal muscle (Jing et al., 2022). Lenvatinib administration also reduced mitochondrial carnitine-related and oxidative phosphorylation (OXPHOS) proteins in the skeletal muscle. Moreover, lenvatinib inhibited protein synthesis and mitochondrial function in cultured C2C12 myocytes in a dose-dependent manner (Jing et al., 2022). In patients with HCC, we found that the plasma acylcarnitine to free carnitine ratio, a marker of carnitine insufficiency, was significantly increased on day 14 of lenvatinib therapy initiation, and that the increase was apparently correlated with the change in fatigue severity on day 28 (Okubo et al., 2020). Furthermore, we showed that the skeletal muscle index, a marker of sarcopenia, was significantly lower in patients with HCC at 6 and 12 weeks of lenvatinib therapy initiation than at baseline, and that the decrease was completely prevented by the co-administration of L-carnitine (Okubo et al., 2021). Taken together, these results strongly suggest that L-carnitine supplementation attenuates lenvatinib-induced muscle toxicity. However, the underlying mechanism and effect of L-carnitine on antineoplastic activity of lenvatinib remain unclear. To address these issues, we confirmed the preventive effect of L-carnitine against lenvatinib-induced muscle toxicity in rats and investigated the impact of L-carnitine on lenvatinib-induced inhibition of protein synthesis and angiogenesis *in vitro*.

## 2 Materials and methods

### 2.1 Rats and treatments

8-week old male Wistar rats (total number = 21; Japan SLC, Hamamatsu, Japan) were housed under controlled temperature (approximately 23°C), humidity (approximately 55%), and light (12 h/12 h light/dark cycle) conditions, and fed a regular diet (CRF-1; Oriental Yeast, Tokyo, Japan) and water *ad libitum* at the Institute for Experimental Animals of Kanazawa University (Kanazawa, Japan). After 1-week acclimation period, the rats were divided into four groups: control (*n* = 5), lenvatinib (*n* = 5), lenvatinib + low-dose L-carnitine (*n* = 5), and lenvatinib + high-dose L-carnitine (*n* = 6). Similar to our previous study (Jing et al., 2022), all groups received once-daily oral dose of lenvatinib mesylate (2 mg/kg; Carbosynth, Berkshire, UK) or vehicle (0.5% w/v methyl cellulose 400 solution; Fujifilm Wako Pure Chemical, Osaka, Japan) at a volume of 1 mL/kg for 14 days. Additionally, L-carnitine (150 or 300 mg/kg; Fujifilm Wako Pure Chemical) or vehicle (distilled water) was co-administered in a volume of 2 mL/kg once daily for 14 days to all rats. The selection of L-carnitine doses was based on the previous studies (Aboubakr et al., 2020), (Rajasekar et al., 2008). Body weight and food intake were measured daily, and the rats were euthanized by cardiac exsanguination under deep anesthesia (using a mixture of medetomidine, midazolam, and butorphanol) to obtain skeletal muscle (gastrocnemius) samples on day 15 of treatment initiation. The obtained samples were flash-frozen in liquid nitrogen and stored at −80°C until use.

All animal protocols were approved by the Institutional Committee for Ethical Use of Experimental Animals (approval no. AP-194096), and performed in accordance with the Guidelines for the Care and Use of Laboratory Animals at Kanazawa University.

### 2.2 Measurement of carnitine

Free L-carnitine content in the skeletal muscle was measured using L-carnitine assay kit (Merck, St. Louis, MO, USA). Briefly, approximately 20 mg of muscle sample was homogenized in 500 μL of carnitine assay buffer and centrifuged at 13,000 × *g* for 10 min. The supernatant (50 μL) was incubated with 50 μL of an appropriate reaction mixture for 30 min at room temperature, and the absorbance at 570 nm was measured.

### 2.3 C2C12 myocytes and treatments

C2C12 mouse myoblast cell line was purchased from RIKEN BRC Cell Bank (Lot No. 41, Tsukuba, Japan) and maintained in Dulbecco’s modified Eagle’s medium (DMEM; Thermo Fisher Scientific, Waltham, MA, USA) supplemented with 10% fetal bovine serum (Biowest, Nuaille, France), 100 μg/mL streptomycin, and 100 U/mL penicillin (Thermo Fisher Scientific) at 37°C in a humidified atmosphere containing 5% CO_2_. Differentiation of the cells to myocytes was induced by incubating the cells in a standard differentiation medium consisting of DMEM with 2% horse serum (Thermo Fisher Scientific) for 6 days, and was confirmed by upregulation of mRNA expression of *Myod1* and *Myog* (Jing et al., 2022). Thereafter, myocytes were treated for 24 h with 1 µM lenvatinib or vehicle (0.1% dimethyl sulfoxide) in combination with varying concentrations (0, 1.6, 6.4, or 25.6 mM) of L-carnitine.

### 2.4 Assessment of mitochondrial function and cell viability

Mitochondrial function was assessed by measuring adenosine triphosphate (ATP) content and mitochondrial membrane potential using ATP Assay Kit-Luminescence and JC-1 MitoMP Detection Kit, respectively, following the manufacturer’s instructions (Dojindo Laboratories, Mashiki, Japan). Protein content was determined using Pierce BCA Protein Assay kit (Thermo Fisher Scientific). ATP content was calculated as nmol/mg of protein. The viability of C2C12 myocytes was evaluated using Cell Counting Kit-8 (Dojindo Laboratories).

### 2.5 Protein synthesis assay

The surface sensing of translation (SUnSET) method (Schmidt et al., 2009) was used for protein synthesis assay. C2C12 myocytes were treated with lenvatinib and/or L-carnitine for 4 h and then cultured in fresh medium with 1 µM puromycin for 30 min. Total protein was collected from the cells, and the amount of puromycin incorporated into newly synthesized protein was determined by western blotting.

### 2.6 Human umbilical vein endothelial cells and tube formation assay

Human umbilical vein endothelial cells (HUVECs) were purchased from Lonza (Walkersville, MD USA) and cultured in Endothelial Cell Basal Medium-2 (EBM-2; Lonza). To investigate VEGFR2 phosphorylation, HUVECs were treated for 24 h with 1 µM lenvatinib or vehicle (0.1% dimethyl sulfoxide) in combination with varying concentrations (0, 1.6, 6.4, or 25.6 mM) of L-carnitine. For the tube formation assay, 50 µL of Matrigel (Corning, Glendale, AZ, USA) was loaded into each well of a 96-well plate, and the plate was incubated at 37°C for 30 min before culture. Cells were seeded at a density of 1.5 × 10^4^ cells/well, and then treated with lenvatinib (0.1 or 1 µM) and/or L-carnitine (1.6, 6.4, or 25.6 mM) for 16 h. Tubular network images were obtained using Olympus CKX53 microscope (Evident, Tokyo, Japan), and ImageJ software (ver. 1.53k; National Institutes of Health, Bethesda, MD, USA) was used to identify and quantify the number, width, and length of the tubes.

### 2.7 Aortic ring assay

The mouse aortic ring assay was conducted according to a previously described method (Baker et al., 2012). In brief, the thoracic aortae of 8-week-old C57BL/6 mice were dissected, cleaned, and cut into rings. The rings were embedded in a collagen mixture (Collagen Gel Culturing Kit; Nitta Gelatin, Osaka, Japan) and incubated at 37°C for 30 min to solidify the collagen mixture. The embedded rings were cultured in Opti-Minimal Essential Medium (Thermo Fisher Scientific) supplemented with 2.5% fetal bovine serum, L-glutamate, 100 μg/mL streptomycin, 100 U/mL penicillin, and 20 ng/mL VEGF (PeproTech, Cranbury, NJ, USA), and treated with lenvatinib (0.1 or 1 µM) and/or L-carnitine (1.6, 6.4, or 25.6 mM) for 7 days. Thereafter, the rings were fixed with 4% paraformaldehyde (Fujifilm Wako Pure Chemical) and stained with PE anti-mouse CD31 antibody (BioLegend, San Diego, CA, USA) to identify newly formed microvessels. Dragonfly confocal microscope system (Oxford Instruments, UK) was used to capture images of the stained rings, and the ImageJ software was used to quantify the number of branches.

### 2.8 Western blotting

Proteins were extracted from the tissue samples, C2C12 myocytes, or HUVECs using RIPA lysis buffer containing protease and phosphatase inhibitor cocktails (Nacalai Tesque, Kyoto, Japan), separated using SDS-PAGE, and transferred to PVDF membranes. The membranes were blocked using Bullet Blocking One for Western Blotting (Nacalai Tesque), incubated overnight at 4°C with the primary antibody, and then incubated with an HRP-conjugated secondary antibody. The antibodies used are listed in Table 1. Finally, the protein bands were imaged using ImageQuant LAS 4000 camera system (GE Healthcare, Chicago, IL, USA), and their intensity was quantified using the ImageJ software.

**TABLE 1:** Antibodies used for Western blotting.

Antibody	Source	Dilution
OCTN2	Proteintech 16331-1-AP	1:1,000
CPT1B	Abcam ab104662	1:1,000
SLC25A20	Abcam ab224388	1:1,000
CPT2	Abcam ab181114	1:1,000
Total OXPHOS Rodent WB Antibody Cocktail	Abcam ab110413	1:1,000
VEGF Receptor 2 (55B11)	Cell Signaling 2479	1:1,000
Phospho-VEGF Receptor 2 (Tyr1175)	Cell Signaling 2478	1:1,000
Puromycin, clone 12D10	Merck MABE343	1:1,000
GAPDH (D16H11), HRP conjugate	Cell Signaling 8884	1:1,000
Rabbit IgG, HRP-linked	Cell Signaling 7074	1:2000
Mouse IgG, HRP-linked	Cell Signaling 7076	1:2000

### 2.9 Statistical analysis

Data are expressed as the mean and standard deviation (SD). Statistical differences were determined using one-way analysis of variance followed by Tukey’s multiple comparison test. GraphPad Prism 9 software (GraphPad Software, San Diego, CA, USA) was used for statistical analyses. Statistical *p*-value <0.05 was considered significant.

## 3 Results

### 3.1 L-carnitine supplementation prevented lenvatinib-induced reduction in L-carnitine content and protein expression in the skeletal muscle of rats

**FIGURE 1:**
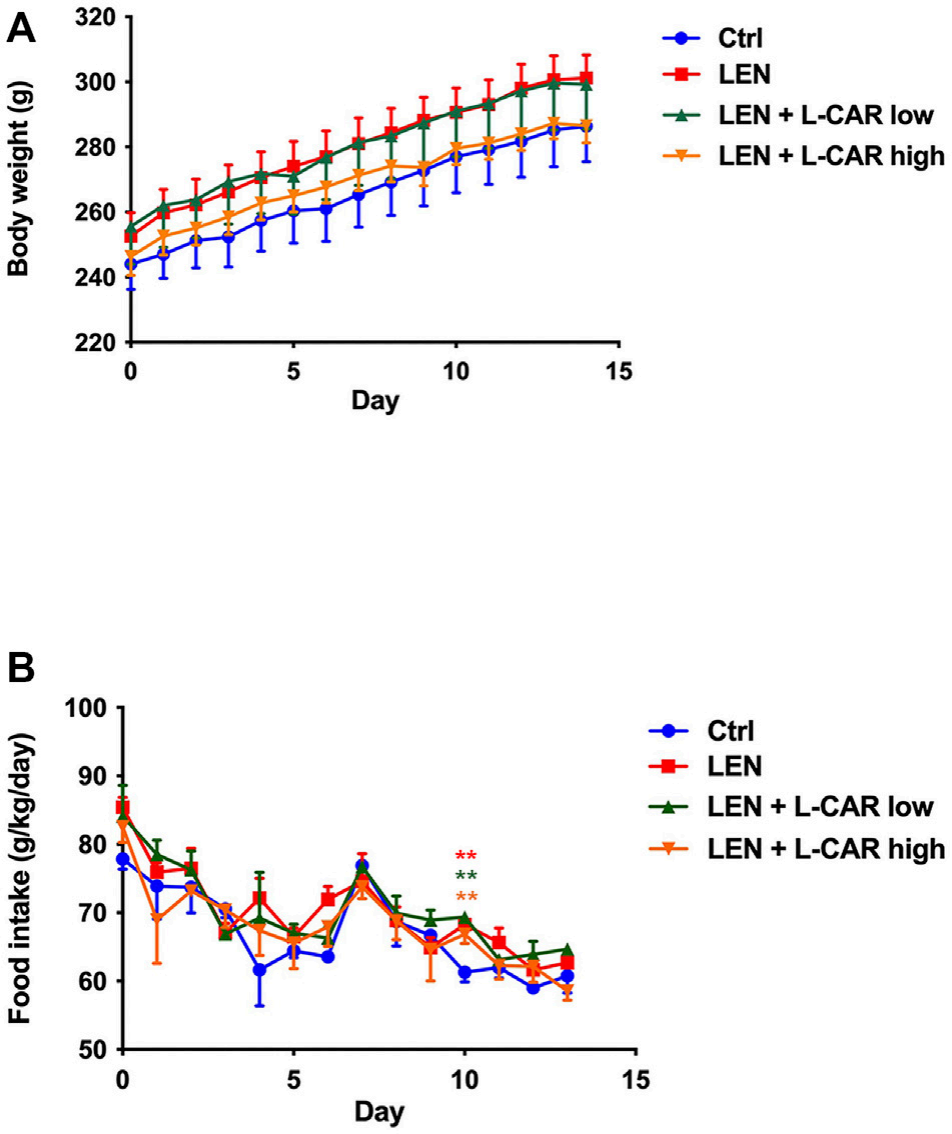
Effects of lenvatinib and L-carnitine supplementation on body weight **(A)** and food intake **(B)**. Lenvatinib mesylate (2 mg/kg), L-carnitine (150 or 300 mg/kg), and/or vehicle were orally administrated once daily for 14 days to rats in the lenvatinib (LEN; *n* = 5), lenvatinib + low-dose L-carnitine (LEN + L-CAR low; *n* = 5), lenvatinib + high-dose L-carnitine (LEN + L-CAR high; *n* = 6), or control (Ctrl) group (*n* = 5), respectively. Data are presented as the mean ± SD. **p < 0.01 *vs*. Ctrl group.

**FIGURE 2:**
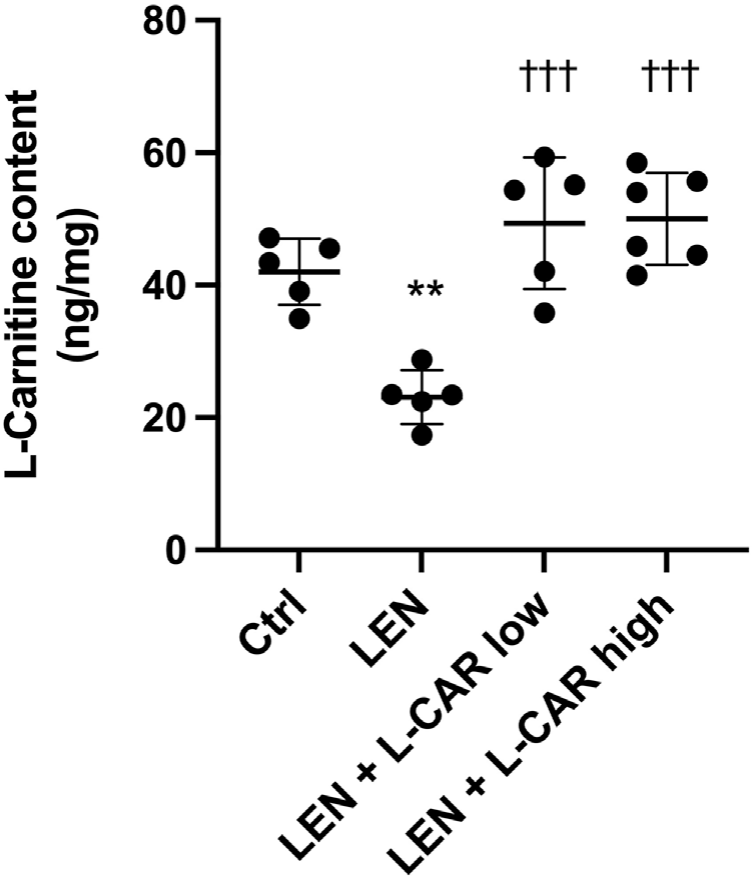
L-Carnitine content in the skeletal muscle after 14-day administration of lenvatinib with or without L-carnitine. Data are presented as the mean +SD of experiments in 5–6 rats. **p < 0.01 *vs*. Ctrl group; ^†††^ *p* < 0.001 vs. LEN group.

**FIGURE 3:**
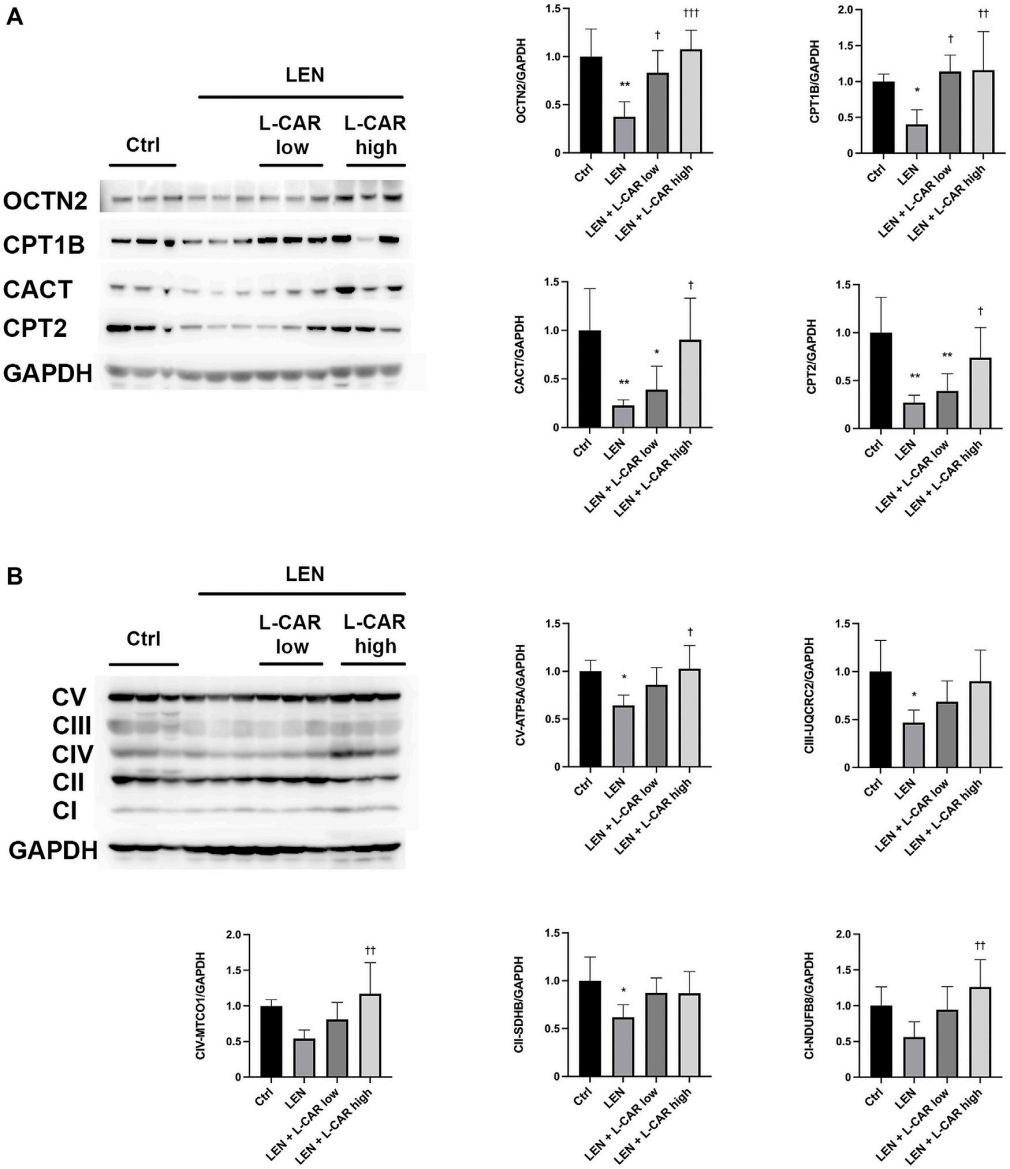
Effects of L-carnitine supplementation on the expression levels of carnitine-related **(A)** and oxidative phosphorylation (OXPHOS) proteins **(B)** in the skeletal muscle of rats treated with lenvatinib. The representative western blot images are shown in the left panel. Data are presented as the mean +SD of experiments in 5–6 rats. *p < 0.05, **p < 0.01 *vs*. Ctrl group; ^†^ *p* < 0.05, ^††^ *p* < 0.01, ^†††^ *p* < 0.001 vs. LEN group.

To investigate whether L-carnitine supplementation prevents lenvatinib-induced reduction in protein expression in the skeletal muscle *in vivo*, we administered lenvatinib with or without L-carnitine once daily for 14 days to rats. These treatments did not obviously alter the food intake or body weight of the rats throughout the treatment period (Figure 1). Consistent with our previous results (Jing et al., 2022), lenvatinib significantly reduced L-carnitine content (Figure 2) and downregulated the expression of carnitine-related (Figure 3A) and OXPHOS proteins (Figure 3B) in the skeletal muscle on day 15 of treatment initiation. These effects of lenvatinib were completely offset by co-administration of high-dose L-carnitine. Interestingly, although L-carnitine content was comparable between the low-dose and high-dose L-carnitine groups (Figure 2), the counteracting effect of L-carnitine on protein expression was, at least partly, greater in the high-dose group than in the low-dose group (Figure 3). This suggests that an excess amount of L-carnitine to the maximum storage is required for effective prevention of lenvatinib-induced adverse effects in the skeletal muscle.

### 3.2 L-carnitine counteracted the adverse effects of lenvatinib on protein expression and mitochondrial function in C2C12 myocytes

**FIGURE 4:**
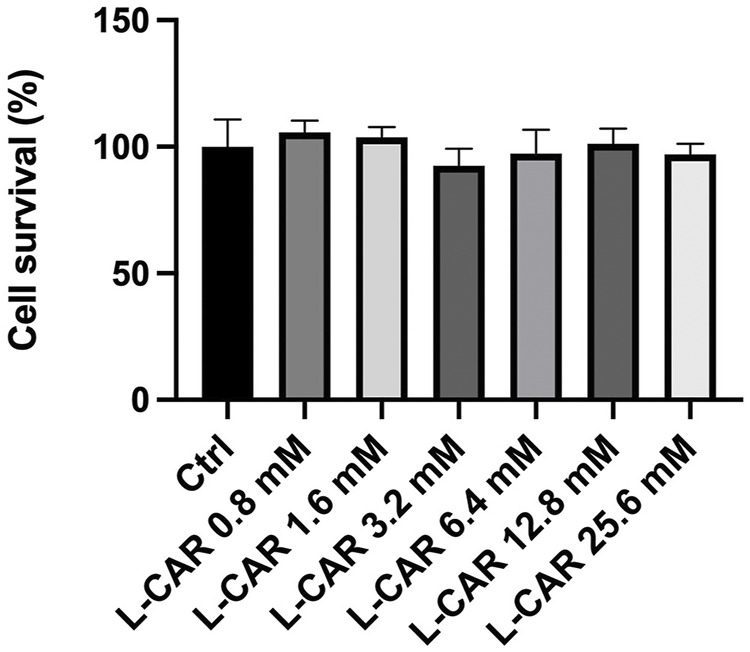
Effect of L-carnitine on cell survival in C2C12 myocytes. Differentiated C2C12 myocytes were treated with different concentrations of L-carnitine for 24 h. Data are presented as the mean +SD (*n* = 6).

**FIGURE 5:**
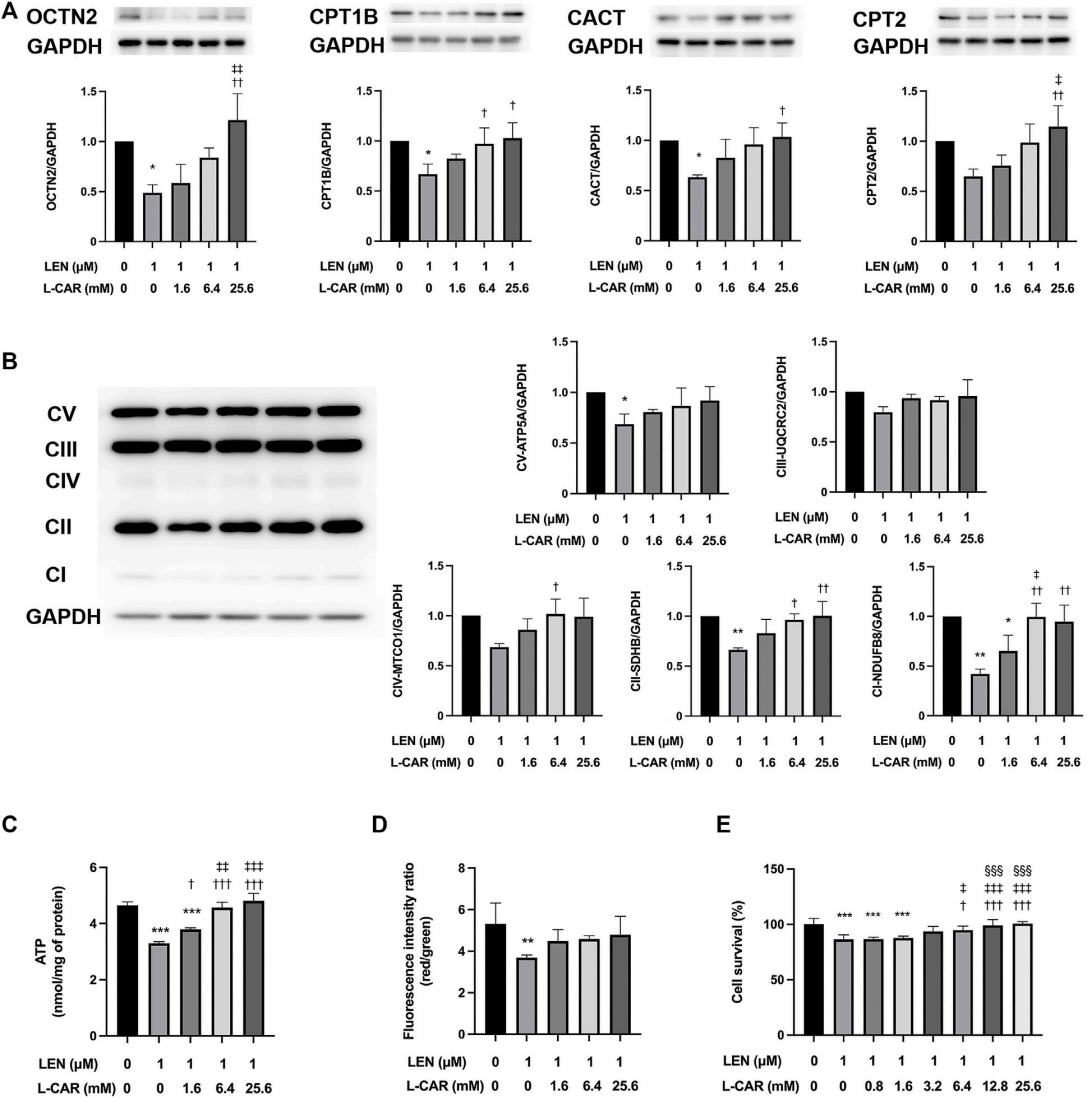
Effects of L-carnitine on carnitine-related and OXPHOS protein expression and mitochondrial function in lenvatinib-treated C2C12 myocytes. Differentiated C2C12 myocytes were treated with lenvatinib (1 µM), L-carnitine (1.6, 6.4, or 25.6 mM), or vehicle for 24 h. Thereafter, the expression levels of carnitine-related **(A)** and oxidative phosphorylation (OXPHOS) proteins **(B)**, adenosine triphosphate (ATP) content **(C)**, mitochondrial membrane potential **(D)**, and cell survival **(E)** were measured in the cells. The upper panel in Fig. A and left panel in Fig. B represent the results of three independent experiments. Data are presented as the mean +SD (*n* = 3 for panels **(A–C)**; *n* = 5 for panel **(D)**; and *n* = 6 for panel **(E)**. *p < 0.05, **p < 0.01, ***p < 0.001 *vs*. Ctrl group; ^†^ *p* < 0.05, ^††^ *p* < 0.01, ^†††^ *p* < 0.001 vs. LEN alone group; ^‡^ *p* < 0.05, ^‡‡^ *p* < 0.01, ^‡‡‡^ *p* < 0.001 vs. LEN + the lowest-dose L-CAR group; ^§§§^ *p* < 0.001 vs. LEN +1.6 mM L-CAR dose group **(E)**.

To further explore the effects of L-carnitine on lenvatinib-induced muscle toxicity, differentiated C2C12 myocytes were exposed to lenvatinib and/or L-carnitine for 24 h. The concentrations (1.6, 6.4, or 25.6 mM) of L-carnitine were selected based on the physiological concentration in human skeletal muscle (approximately 4.5 mM) (Evans and Fornasini, 2003) and a preliminary experiment indicating non-cytotoxicity at up to 25.6 mM (Figure 4). As we reported previously (Jing et al., 2022), lenvatinib at 1 μM significantly decreased the expression of carnitine-related (Figure 5A) and OXPHOS proteins (Figure 5B), along with reduction in ATP content (Figure 5C) and mitochondrial membrane potential (Figure 5D), which are markers of mitochondrial function (Castilla-Cortázar et al., 2011), and cell survival (Figure 5E). Consistent with the findings in rats (Figures 3A, B), L-carnitine counteracted lenvatinib-induced decrease in protein expression in a dose-dependent manner (Figures 5A, B). Moreover, high-dose L-carnitine supplementation completely prevented the reduction in mitochondrial function markers (Figures 5C, D), and ultimately attenuated cytotoxicity (Figure 5E) in lenvatinib-treated C2C12 myocytes.

### 3.3 L-carnitine counteracted lenvatinib-induced protein synthesis inhibition in C2C12 myocytes

**FIGURE 6:**
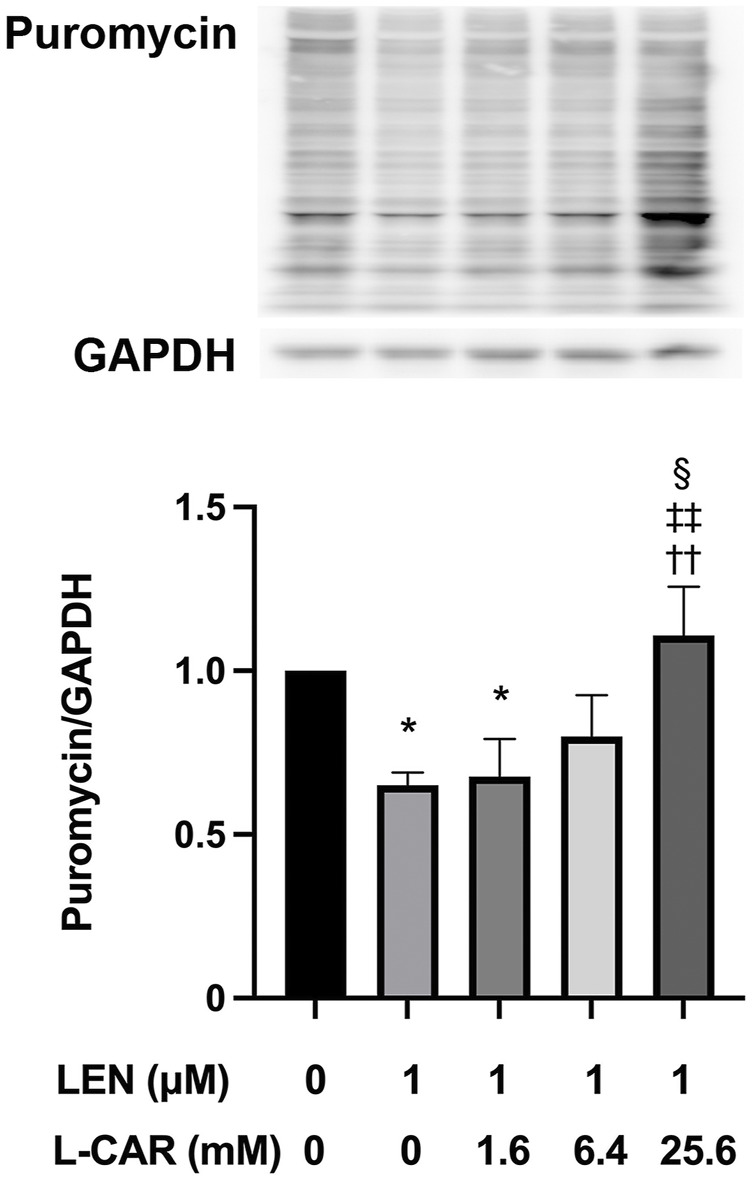
Effect of L-carnitine on protein synthesis in lenvatinib-treated C2C12 myocytes. The cells were treated with lenvatinib (1 µM), L-carnitine (1.6, 6.4, or 25.6 mM), or vehicle for 4 h. Thereafter, the rate of protein synthesis was assessed using the SUnSET method. The upper panel represents the results, and the lower panel presents data as the mean +SD of three independent experiments. *p < 0.05 *vs*. Ctrl group; ^††^ *p* < 0.01 vs. LEN alone group; ^‡‡^ *p* < 0.01 vs. LEN +1.6 mM L-CAR group; ^§^ *p* < 0.05 vs. LEN +6.4 mM L-CAR dose group.

We have shown that lenvatinib inhibits global protein synthesis in C2C12 myocytes at concentrations ≥100 nM, which is equivalent to the trough plasma concentration in patients with HCC (Okubo et al., 2022), in C2C12 myocytes (Jing et al., 2022). In preliminary experiments, L-carnitine appeared to counteract the lenvatinib (1 μM)-induced inhibition of protein synthesis after 1–24 h of exposure (Supplementary Figure S1). This counteracting effect of L-carnitine was dose-dependent and statistically significant at least at 4 h of exposure (Figure 6). Moreover, this effect of L-carnitine on protein synthesis was consistent with that of protein expression, mitochondrial function, and cell survival (Figure 5), suggesting that these deleterious effects of lenvatinib were primarily caused by its inhibitory action on protein synthesis.

### 3.4 L-carnitine had no influence on anti-angiogenic efficacy of lenvatinib

**FIGURE 7:**
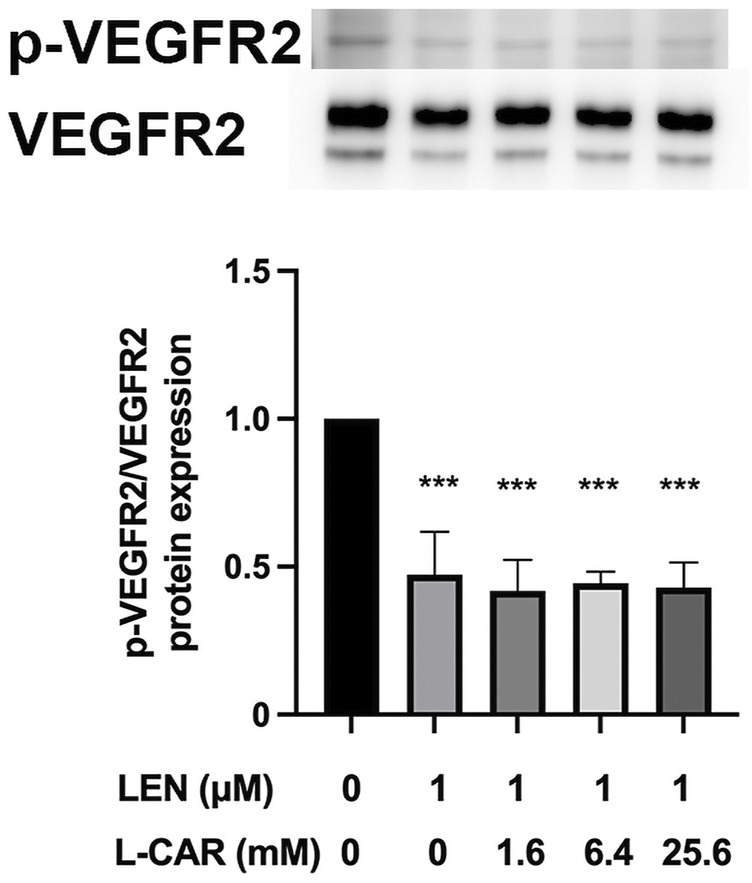
Effect of L-carnitine on phosphorylation of vascular endothelial growth factor receptor (VEGFR) 2 in lenvatinib-treated human umbilical vein endothelial cells (HUVECs). The cells were treated with lenvatinib (1 µM), L-carnitine (1.6, 6.4, or 25.6 mM), or vehicle for 24 h. The upper panel represents the results, and the lower panel presents data as the mean +SD of three independent experiments. ***p < 0.001 *vs*. Ctrl group.

**FIGURE 8:**
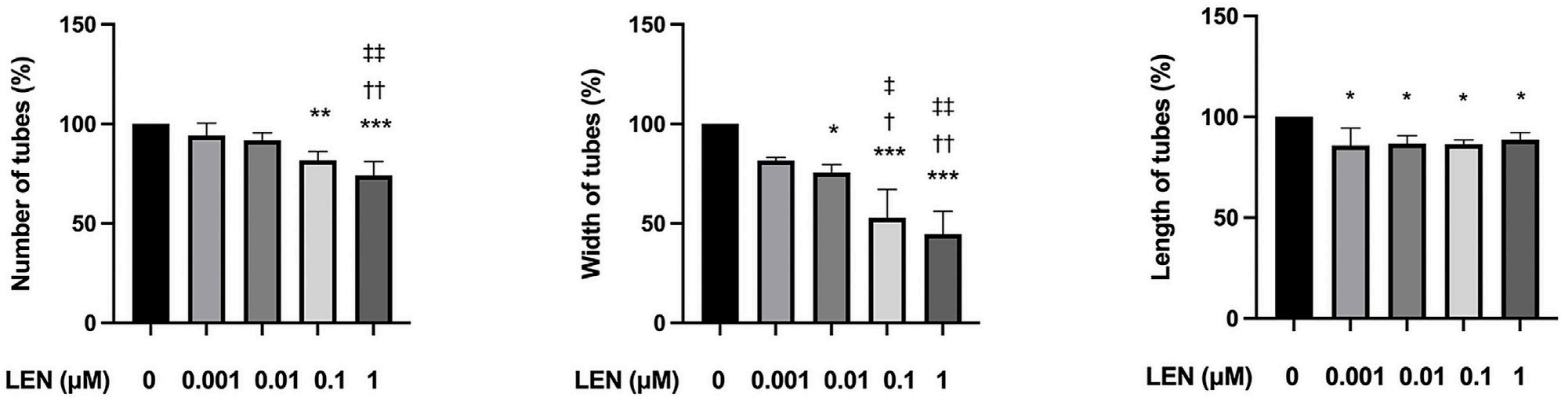
Effect of lenvatinib on tube formation in HUVECs. The cells were treated with different concentrations of lenvatinib for 16 h. Data are presented as the mean +SD (*n* = 3 from three independent experiments). *p < 0.05, **p < 0.01, ***p < 0.001 *vs*. Ctrl group; ^†^ *p* < 0.05, ^††^ *p* < 0.01 vs. 0.001 µM LEN group; ^‡^ *p* < 0.05, ^‡‡^ *p* < 0.01 vs. 0.01 µM LEN group.

**FIGURE 9:**
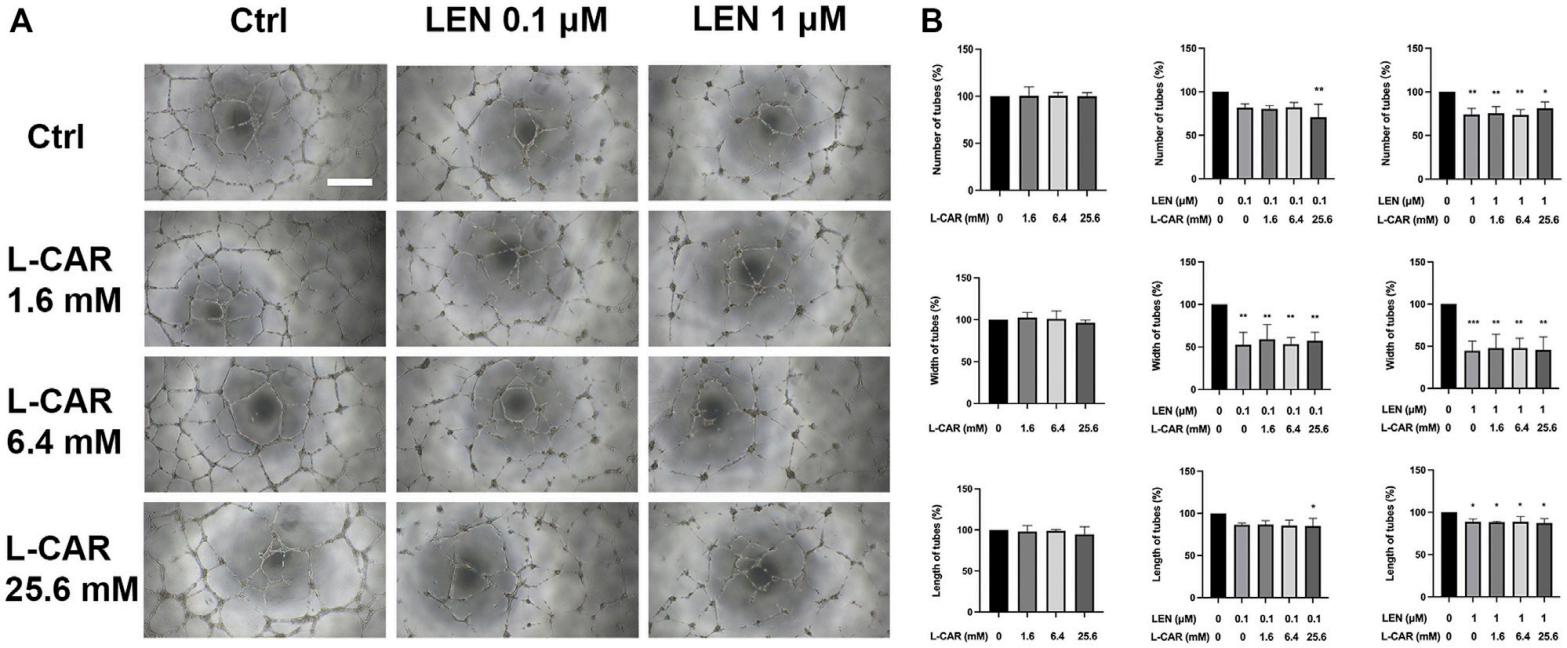
Effects of lenvatinib and/or L-carnitine on tube formation in HUVECs. The cells were treated with lenvatinib (0.1 or 1 µM), L-carnitine (1.6, 6.4, or 25.6 mM), and/or vehicle for 16 h. Thereafter, the number, width, and length of tubes in each well were measured using the ImageJ software. For analyses of width and length, the mean of the three maximum values for each well was used. **(A)** Representative images of tube formation. The white bar indicates 0.5 mm. **(B)** Data are presented as the mean +SD (*n* = 3 from three independent experiments). *p < 0.05, **p < 0.01 *vs*. Ctrl group.

The above findings clearly indicate that L-carnitine supplementation prevents lenvatinib-induced muscle toxicity. To further verify the clinical application of the combination, we investigated the impact of L-carnitine on anti-angiogenic effect, the principal action, of lenvatinib *in vitro*. As shown in Figure 7, 1 µM lenvatinib significantly inhibited the phosphorylation of VEGFR2, one of its target molecules (Matsui et al., 2008), in HUVECs. This inhibition was not prevented by L-carnitine at up to 25.6 mM. In the tube formation assay using HUVECs, which is a well-established, quantitative, and reliable angiogenesis assay (Arnaoutova and Kleinman, 2010), the inhibitory effects of lenvatinib were dose-dependent and significant at 0.1 and 1 µM (Figure 8). In contrast, L-carnitine did not affect any of the parameters determined (number, width, and length of tubes), even at the highest concentration (25.6 mM) (Figure 9). Consistent with the results of VEGFR2 phosphorylation, L-carnitine (up to 25.6 mM) did not affect the values of these parameters (Figure 9). Similarly, L-carnitine did not inhibit the effects of lenvatinib at lower concentration (0.1 µM).

**FIGURE 10:**
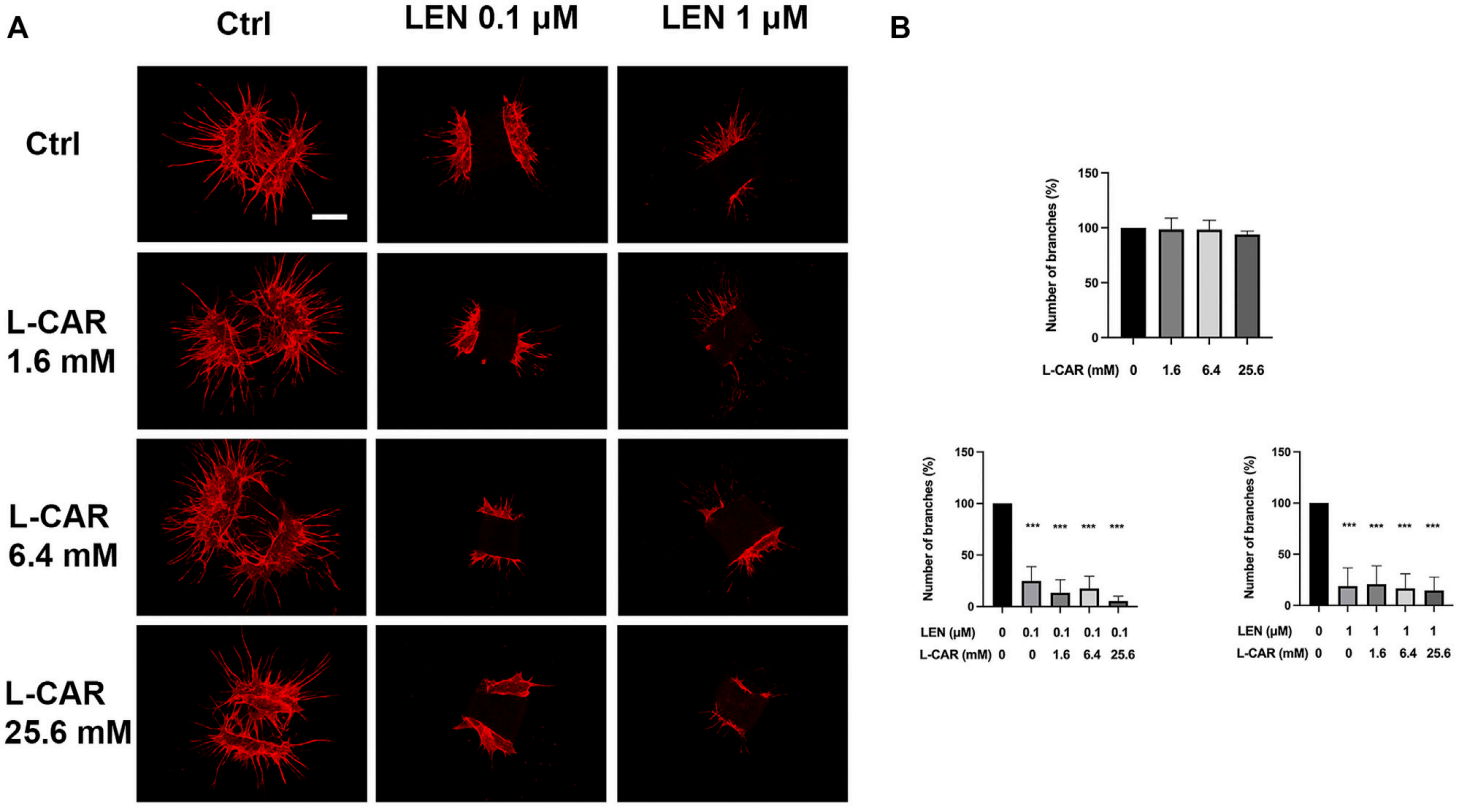
Effects of lenvatinib and/or L-carnitine on branch formation in mouse aortic ring assay. Aortic rings embedded in a collagen matrix were treated with lenvatinib (0.1 or 1 µM), L-carnitine (1.6, 6.4, or 25.6 mM), and/or vehicle for 7 days. Thereafter, the rings were cultured without any treatment for another 6 days. The ImageJ software was used to identify and quantify the number of branches. **(A)** Representative images of tube formation. The white bar indicates 0.5 mm. **(B)** Data are presented as the mean +SD (*n* = 3 from three independent experiments). ***p < 0.001 *vs*. Ctrl group.

To investigate the effect of L-carnitine supplementation on angiogenesis more physiologically (Baker et al., 2012), we further conducted mouse aortic ring assay. As shown in Figure 10, L-carnitine (up to 25.6 mM) did not influence VEGF-stimulated angiogenesis or the inhibitory effect of lenvatinib. These results indicated that L-carnitine, even at a high dose, had a minimal effect on anti-angiogenic action of lenvatinib.

## 4 Discussion

Lenvatinib therapy is a standard treatment for various cancers but frequently causes adverse reactions, including carnitine insufficiency (Okubo et al., 2020) and sarcopenia (Okubo et al., 2021). The study findings confirmed that lenvatinib reduced the carnitine content and mitochondrial protein expression in the skeletal muscle of rats, and that these effects were completely prevented by L-carnitine supplementation. Moreover, in C2C12 myocytes, L-carnitine counteracted lenvatinib-induced protein synthesis inhibition, mitochondrial dysfunction, and cell toxicity. Thus, these results suggest that lenvatinib exhibits muscle toxicity, at least partly, due to carnitine shortage, and that this adverse effect can be prevented by L-carnitine supplementation.

L-Carnitine has excellent tolerability (Evans and Fornasini, 2003) and long been used in healthy people and patients. For example, many studies have shown its usefulness for recovery from exercise in athletes and active people and for the improvement of muscle mass and function (i.e., sarcopenia) in elderly people (Fielding et al., 2018). Moreover, L-carnitine supplementation increases lean body mass in patients with advanced cancer (Gramignano et al., 2006), (Madeddu et al., 2012) and muscle mass in patients with liver cirrhosis (Hiramatsu et al., 2019). These beneficial effects of L-carnitine on skeletal muscle are considered attributed to both inhibition of pathophysiological conditions (inflammation, oxidative stress, mitochondrial dysfunction, myonuclear apoptosis, and proteolysis) and promotion of protein synthesis (Ringseis et al., 2013). Hence, it is likely that all these actions of L-carnitine contribute to the preventive effect against lenvatinib-induced muscle toxicity.

Non-etheless, the effect of L-carnitine on protein synthesis seems to be the primary mechanism for protection against lenvatinib-induced muscle toxicity. The skeletal muscle cannot synthesize L-carnitine despite its high demand (Evans and Fornasini, 2003) and depends on its uptake from the extracellular fluid through OCTN2 (Tamai, 2013). Therefore, lenvatinib might have decreased the L-carnitine content by reducing OCTN2 expression in the skeletal muscle of rats. Our previous study demonstrated that this reduction was not caused at the mRNA level, but at the protein level, in both skeletal muscle and C2C12 myocytes (Jing et al., 2022). Moreover, in this study, L-carnitine supplementation prevented downregulation of OCTN2 protein expression *in vivo* and *in vitro*. Taken together, the action of L-carnitine on OCTN2 translation alone may be sufficient to explain its preventive effect against lenvatinib-induced muscle toxicity.

The molecular mechanism underlying lenvatinib-mediated inhibition of protein synthesis remains unclear. However, sorafenib, another TKI, has been reported to suppress translation initiation *via* inhibition of the mechanistic target of rapamycin (mTOR) signaling (Sauzay et al., 2018). Sorafenib inhibits the phosphorylation of the eukaryotic translation initiation factor 4E-binding protein 1, a key negative regulator of translation controlled by mTOR (Sauzay et al., 2018), (Rosenberg et al., 2018). Because VEGFRs and FGFRs, targets of lenvatinib, exert various physiological effects partly through the mTOR signaling (Liu et al., 2021), it is plausible that lenvatinib also inhibits protein synthesis *via* the mTOR pathway. In contrast, L-carnitine has been shown to activate mTOR signaling in the skeletal muscle of rats (Keller et al., 2013) and piglets (Madsen et al., 2018). Insulin-like growth factor 1 (IGF1) is a key growth factor that increases protein synthesis *via* the phosphoinositide 3-kinase (PI3K)/Akt/mTOR pathway (Yoshida and Delafontaine, 2020). L-Carnitine supplementation in rats reportedly increased both plasma concentration and mRNA expression of IGF1 in the liver, the major tissue producing circulating IGF1 (Keller et al., 2013), (Heo et al., 2001). Since studies using conditional knockout mice indicate that muscle, but not hepatic, IGF1 is a critical factor for skeletal growth (Bikle et al., 2015), it is possible that L-carnitine activates the mTOR signaling by increasing local IGF1 production. However, L-carnitine appeared to begin to exert its effect within 1 h of exposure in C2C12 cells (Supplementary Figure S1), suggesting that L-carnitine directly affects the mTOR pathway. Further studies are required to clarify the molecular mechanisms underlying the effects of lenvatinib and L-carnitine on protein synthesis.

The PI3K/Akt/mTOR signaling pathway is commonly involved in cell survival, growth, and proliferation in human cancers (Peng et al., 2022). In addition, CPTs not only provide energy for the growth and metastasis of tumor cells by activating β-oxidation but also interact with other cellular signaling pathways involved in cancer pathogenesis (Wang et al., 2021). Thus, L-carnitine supplementation appears to promote cancer progression. On the other hand, angiogenesis, the formation of new blood vessels, is also essential for the proliferation, growth, and metastasis of tumor cells (Hanahan and Folkman, 1996). In addition, VEGF/VEGFR2 signaling plays a critical role in tumor angiogenesis (Shah et al., 2020). In this study, we confirmed the inhibitory effect of lenvatinib on VEGFR2 phosphorylation, and observed that L-carnitine had no effect on this action in HUVECs. Moreover, simultaneous addition of L-carnitine did not disrupt lenvatinib-induced inhibition of angiogenesis in both tube formation and aortic ring assays. These results clearly indicate that L-carnitine does not counteract the inhibitory effect of lenvatinib on the angiogenic signaling pathway(s), at least *in vitro*. Taken together, it remains unclear whether L-carnitine reduces the “anticancer” effect of lenvatinib. Reportedly, VEGF signaling increases VEGFR2 expression and phosphorylation, and stimulates proliferation in VEGF-stimulated C2C12 cells (Sassoli et al., 2012). However, VEGFR2 protein was not detected under the non-stimulated conditions used in this study (Supplementary Figure S2), and this might potentially reduce the effects of lenvatinib and L-carnitine in C2C12 cells. Because the microenvironment differs between *in vitro* and *in vivo* conditions and between healthy and cancer individuals, the results of this study should be confirmed in *in vivo* cancer models.

## 5 Conclusion

In conclusion, L-carnitine supplementation completely prevented lenvatinib-induced downregulation of carnitine-related and OXPHOS proteins in the skeletal muscle of rats and C2C12 myocytes. Moreover, L-carnitine counteracted the inhibitory action of lenvatinib on global protein synthesis in C2C12 myocytes, the major mechanism considered for preventive effect of L-carnitine against lenvatinib-induced muscle toxicity. In contrast, L-carnitine had no influence on anti-angiogenic effect, the principal action, of lenvatinib. We have previously demonstrated the beneficial effects of L-carnitine supplementation on fatigue (Okubo et al., 2020) and sarcopenia (Okubo et al., 2021) in patients with HCC treated with lenvatinib. In addition, L-carnitine has been used as a supplement in patients with cancer. Further studies are needed to investigate the effects of L-carnitine supplementation on treatment continuation, tumor progression, and ultimately, mortality in patients treated with lenvatinib.

## Data Availability

The raw data supporting the conclusions of this article will be made available by the authors, without undue reservation.

## References

[B1] Aboubakr M. Elsayd F. Soliman A. Fadl S. E. El-Shafey A. Abdelhiee E. Y. (2020). l-Carnitine and vitamin E ameliorate cardiotoxicity induced by tilmicosin in rats. Environ. Sci. Pollut. Res. 27, 23026–23034. 10.1007/s11356-020-08919-6 32329006

[B2] Arnaoutova I. Kleinman H. K. (2010). *In vitro* angiogenesis: Endothelial cell tube formation on gelled basement membrane extract. Nat. Protoc. 5, 628–635. 10.1038/nprot.2010.6 20224563

[B3] Baker M. Robinson S. D. Lechertier T. Barber P. R. Tavora B. D’Amico G. (2012). Use of the mouse aortic ring assay to study angiogenesis. Nat. Protoc. 7, 89–104. 10.1038/nprot.2011.435 22193302

[B4] Bartlett K. Eaton S. (2004). Mitochondrial β-oxidation. Eur. J. Biochem. 271, 462–469. 10.1046/j.1432-1033.2003.03947.x 14728673

[B5] Bikle D. D. Tahimic C. Chang W. Wang Y. Philippou A. Barton E. R. (2015). Role of IGF-I signaling in muscle bone interactions. Bone 80, 79–88. 10.1016/j.bone.2015.04.036 26453498 PMC4600536

[B6] Castilla-Cortázar I. García-Fernández M. Delgado G. Puche J. E. Sierra I. Barhoum R. (2011). Hepatoprotection and neuroprotection induced by low doses of IGF-II in aging rats. J. Transl. Med. 9, 103. 10.1186/1479-5876-9-103 21733157 PMC3150260

[B7] Evans A. M. Fornasini G. (2003). Pharmacokinetics of L-carnitine. Clin. Pharmacokinet. 42, 941–967. 10.2165/00003088-200342110-00002 12908852

[B8] Fielding R. Riede L. Lugo J. P. Bellamine A. (2018). L-carnitine supplementation in recovery after exercise. Nutrients 10, 349–417. 10.3390/nu10030349 29534031 PMC5872767

[B9] Gramignano G. Lusso M. R. Madeddu C. Massa E. Serpe R. Deiana L. (2006). Efficacy of l-carnitine administration on fatigue, nutritional status, oxidative stress, and related quality of life in 12 advanced cancer patients undergoing anticancer therapy. Nutrition 22, 136–145. 10.1016/j.nut.2005.06.003 16459226

[B10] Hanahan D. Folkman J. (1996). Patterns and emerging mechanisms of the angiogenic switch during tumorigenesis. Cell 86, 353–364. 10.1016/S0092-8674(00)80108-7 8756718

[B11] Heo Y. R. Kang C. W. Cha Y. S. (2001). L-Carnitine changes the levels of insulin-like growth factors (IGFs) and IGF binding proteins in streptozotocin-induced diabetic rat. J. Nutr. Sci. Vitaminol. (Tokyo) 47, 329–334. 10.3177/jnsv.47.329 11814147

[B12] Hiramatsu A. Aikata H. Uchikawa S. Ohya K. Kodama K. Nishida Y. (2019). Levocarnitine use is associated with improvement in sarcopenia in patients with liver cirrhosis. Hepatol. Commun. 3, 348–355. 10.1002/hep4.1309 30859147 PMC6396356

[B13] Jing Z. Okubo H. Morishige J. Xu P. Hasan N. Nagata N. (2022). Lenvatinib causes reduced expression of carnitine/organic cation transporter 2 and carnitine deficiency in the skeletal muscle of rats. Toxicol. Lett. 366, 17–25. 10.1016/j.toxlet.2022.06.012 35788046

[B14] Keller J. Couturier A. Haferkamp M. Most E. Eder K. (2013). Supplementation of carnitine leads to an activation of the IGF-1/PI3K/Akt signalling pathway and down regulates the E3 ligase MuRF1 in skeletal muscle of rats. Nutr. Metab. 10, 28–12. 10.1186/1743-7075-10-28 PMC3631133 23497226

[B15] Kim B. H. Yu S. J. Kang W. Cho S. B. Park S. Y. Kim S. U. (2021). Expert consensus on the management of adverse events in patients receiving lenvatinib for hepatocellular carcinoma. J. Gastroenterol. Hepatol. 37, 428–439. 10.1111/jgh.15727 34725855 PMC9299126

[B16] Kudo M. Finn R. S. Qin S. Han K. H. Ikeda K. Piscaglia F. (2018). Lenvatinib versus sorafenib in first-line treatment of patients with unresectable hepatocellular carcinoma: A randomised phase 3 non-inferiority trial. Lancet 391, 1163–1173. 10.1016/S0140-6736(18)30207-1 29433850

[B17] Liu G. Chen T. Ding Z. Wang Y. Wei Y. Wei X. (2021). Inhibition of FGF-FGFR and VEGF-VEGFR signalling in cancer treatment. Cell Prolif. 54, e13009–e13026. 10.1111/cpr.13009 33655556 PMC8016646

[B18] Longo N. Frigeni M. Pasquali M. (2016). Carnitine transport and fatty acid oxidation. Biochim. Biophys. Acta - Mol. Cell Res. 1863, 2422–2435. 10.1016/j.bbamcr.2016.01.023 PMC4967041 26828774

[B19] Madeddu C. Dessì M. Panzone F. Serpe R. Antoni G. Cau M. C. (2012). Randomized phase III clinical trial of a combined treatment with carnitine + celecoxib ± megestrol acetate for patients with cancer-related anorexia/cachexia syndrome. Clin. Nutr. 31, 176–182. 10.1016/j.clnu.2011.10.005 22047681

[B20] Madsen J. G. Seoni E. Kreuzer M. Silacci P. Bee G. (2018). Influence of l-carnitine and l-arginine on protein synthesis and maturation of the semitendinosus muscle of lightweight piglets. J. Anim. Physiol. Anim. Nutr. Berl. 102, 440–451. 10.1111/jpn.12765 28771840

[B21] Matsui J. Yamamoto Y. Funahashi Y. Tsuruoka A. Watanabe T. Wakabayashi T. (2008). E7080, a novel inhibitor that targets multiple kinases, has potent antitumor activities against stem cell factor producing human small cell lung cancer H146, based on angiogenesis inhibition. Int. J. Cancer 122, 664–671. 10.1002/ijc.23131 17943726

[B22] Motzer R. J. Taylor M. H. Evans T. R. J. Okusaka T. Glen H. Lubiniecki G. M. (2022). Lenvatinib dose, efficacy, and safety in the treatment of multiple malignancies. Expert Rev. Anticancer Ther. 22, 383–400. 10.1080/14737140.2022.2039123 35260027 PMC9484451

[B23] Okubo H. Ando H. Ishizuka K. Kitagawa R. Okubo S. Saito H. (2020). Carnitine insufficiency is associated with fatigue during lenvatinib treatment in patients with hepatocellular carcinoma. PLoS One 15, 02297722–e229812. 10.1371/journal.pone.0229772 PMC7053710 32126131

[B24] Okubo H. Ando H. Ishizuka K. Morishige J. Ikejima K. Shiina S. (2022). Impact of genetic polymorphisms on the pharmacokinetics and pharmacodynamics of lenvatinib in patients with hepatocellular carcinoma. J. Pharmacol. Sci. 148, 6–13. 10.1016/j.jphs.2021.08.011 34924131

[B25] Okubo H. Ando H. Nakadera E. Ikejima K. Shiina S. Nagahara A. (2021). Levocarnitine supplementation suppresses lenvatinib-related sarcopenia in hepatocellular carcinoma patients: Results of a propensity score analysis. Nutrients 13, 4428. 10.3390/NU13124428 34959980 PMC8705344

[B26] Peng Y. Wang Y. Zhou C. Mei W. Zeng C. (2022). PI3K/Akt/mTOR pathway and its role in cancer therapeutics: Are we making headway? Front. Oncol. 12, 819128–819217. 10.3389/fonc.2022.819128 35402264 PMC8987494

[B27] Rajasekar P. Viswanathan P. Anuradha C. V. (2008). Renoprotective action of L-carnitine in fructose-induced metabolic syndrome. Diabetes, Obes. Metab. 10, 171–180. 10.1111/j.1463-1326.2007.00825.x 18093214

[B28] Ringseis R. Keller J. Eder K. (2013). Mechanisms underlying the anti-wasting effect of l-carnitine supplementation under pathologic conditions: Evidence from experimental and clinical studies. Eur. J. Nutr. 52, 1421–1442. 10.1007/s00394-013-0511-0 23508457

[B29] Rinninella E. Cintoni M. Raoul P. Pozzo C. Strippoli A. Ponziani F. R. (2020). Skeletal muscle loss during multikinase inhibitors therapy: Molecular pathways, clinical implications, and nutritional challenges. Nutrients 12, 3101–3117. 10.3390/nu12103101 33053632 PMC7601327

[B30] Rosenberg L. Yoon C. H. Sharma G. Bertagnolli M. M. Cho N. L. (2018). Sorafenib inhibits proliferation and invasion in desmoid-derived cells by targeting Ras/MEK/ERK and PI3K/Akt/mTOR pathways. Carcinogenesis 39, 681–688. 10.1093/carcin/bgy038 29538717

[B31] Sassoli C. Pini A. Chellini F. Mazzanti B. Nistri S. Nosi D. (2012). Bone marrow mesenchymal stromal cells stimulate skeletal myoblast proliferation through the paracrine release of VEGF. PLoS One 7, e37512. 10.1371/journal.pone.0037512 22815682 PMC3398011

[B32] Sauzay C. Louandre C. Bodeau S. Anglade F. Godin C. Saidak Z. (2018). Protein biosynthesis, a target of sorafenib, interferes with the unfolded protein response (UPR) and ferroptosis in hepatocellular carcinoma cells. Oncotarget 9, 8400–8414. 10.18632/oncotarget.23843 29492203 PMC5823558

[B33] Schmidt E. K. Clavarino G. Ceppi M. Pierre P. (2009). SUnSET, a nonradioactive method to monitor protein synthesis. Nat. Methods 64, 275–277. 10.1038/nmeth.1314 19305406

[B34] Shah A. A. Kamal M. A. Akhtar S. (2020). Tumor angiogenesis and VEGFR-2: Mechanism, pathways and current biological therapeutic interventions. Curr. Drug Metab. 22, 50–59. 10.2174/1389200221666201019143252 33076807

[B35] Tamai I. (2013). Pharmacological and pathophysiological roles of carnitine/organic cation transporters (OCTNs: SLC22A4, SLC22A5 and Slc22a21). Biopharm. Drug Dispos. 34, 29–44. 10.1002/BDD.1816 22952014

[B36] Wang J. Xiang H. Lu Y. Wu T. Ji G. (2021). The role and therapeutic implication of CPTs in fatty acid oxidation and cancers progression. Am. J. Cancer Res. 11, 2477–2494. 34249411 PMC8263643

[B37] Yoshida T. Delafontaine P. (2020). Mechanisms of IGF-1-mediated regulation of skeletal muscle hypertrophy and atrophy. Cells 9, 1970–2025. 10.3390/cells9091970 32858949 PMC7564605

