# Supplementary material for: L-carnitine prevents lenvatinib-induced muscle toxicity without impairment of the anti-angiogenic efficacy
Source: Front Pharmacol. 2023 Apr 6;14:1182788. doi: 10.3389/fphar.2023.1182788 (PMC10116043; doi:10.3389/fphar.2023.1182788)
Supplement: Supplementary file 1 [file DataSheet1.docx]

Supplementary Material

L-Carnitine prevents lenvatinib-induced muscle toxicity without impairment of the anti-angiogenic efficacy

Zheng Jing, Tomohiro Iba, Hisamichi Naito, Pingping Xu, Jun-ichi Morishige, Naoto Nagata, Hironao Okubo, Hitoshi Ando^*^

*** Correspondence:** Hitoshi Ando: h-ando@med.kanazawa-u.ac.jp

Supplementary Figure 1

Supplementary Figure 2


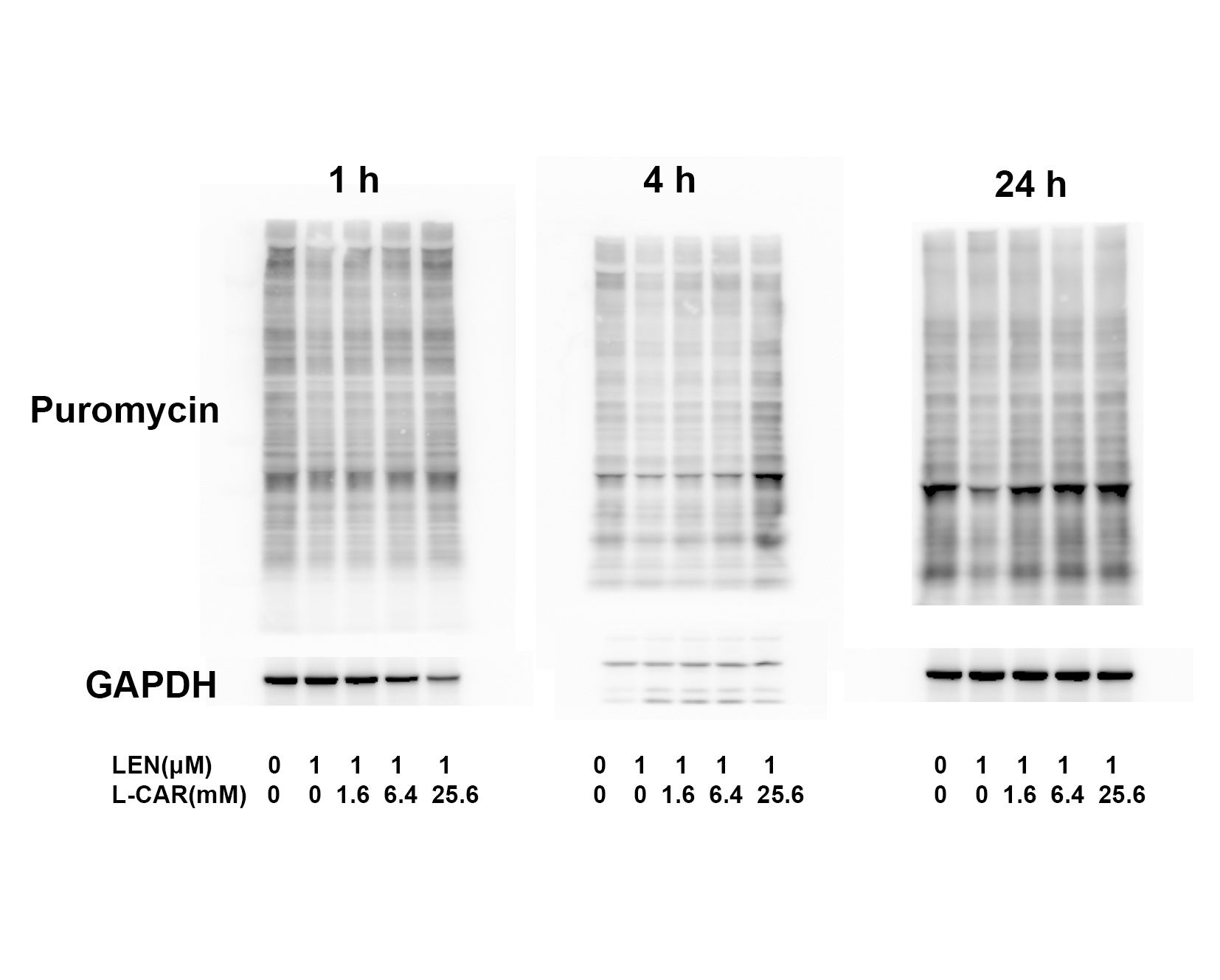


**Supplementary Figure 1.** Effect of L-carnitine on protein synthesis in lenvatinib-treated C2C12 myocytes. The cells were treated with lenvatinib (1 µM), L-carnitine (1.6, 6.4, or 25.6 mM), or vehicle for 1, 4, or 24 h. Thereafter, the rate of protein synthesis was assessed using the SUnSET method.


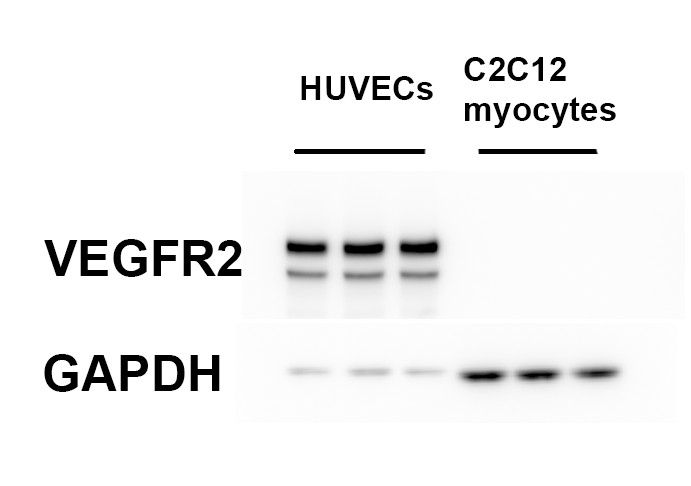


**Supplementary Figure 2.** Expression of vascular endothelial growth factor receptor (VEGFR) 2 in human umbilical vein endothelial cells (HUVECs) and C2C12 myocytes.
